# A multi-method exploration of disgust in eating disorders: Findings and implications for research and treatment

**DOI:** 10.1192/j.eurpsy.2026.10230

**Published:** 2026-07-31

**Authors:** S. Bektas

**Affiliations:** Department of Psychology, Hacettepe University, Ankara, Türkiye

## Abstract

**Introduction:**

Disgust is a multifaceted aversive emotion. Triggers for disgust reactions extend beyond pathogen-related elicitors, encompassing a wide range of stimuli. Anxiety disorders, obsessive-compulsive disorder, and posttraumatic stress disorder can change the perception of disgust. However, despite growing interest in the different types of disgust and the role of disgust for psychiatric disorders, research of its relevance in eating disorders (EDs) remains limited.

**Objectives:**

Three studies conducted in the UK between 2022 and 2025 aimed to examine: 1) differences in disgust conditioning between individuals with EDs and healthy controls, 2) relevance of food disgust to attentional and behavioural patterns and ED symptom severity in people with anorexia nervosa (AN), and 3) differences in characteristics of autobiographical memory and future thinking in response to moral disgust-related cues in individuals with EDs and healthy controls.

**Methods:**

A multi-method approach was applied across three studies: (1) a pilot study adapted an app-based fear learning paradigm to remotely deliver a Pavlovian disgust conditioning task; (2) a virtual reality (VR) study randomly assigned individuals with AN to one of three virtual kitchen scenarios: (a) virtual kitchen only, (b) virtual kitchen + pet, or (c) virtual kitchen + avatar. Food-specific trait disgust was assessed before VR exposure, and food-specific state disgust was assessed afterward. Food interactions, including eye gaze and touching of virtual food items, were also recorded during VR exposure; and (3) a cross-sectional study examined whether the proportion and vividness of specific autobiographical memories or future events in response to moral disgust-related cues (versus neutral cues) differed between individuals with EDs and healthy controls.

**Results:**

The data provided some support for alterations in disgust experiences and their relevance to the psychopathology of EDs. Individuals with EDs reported higher levels of food disgust and self-disgust compared to healthy controls. Altered disgust extinction may be a mechanism underlying ED psychopathology. Despite non-significant differences in the specificity or vividness of memories and future events, individuals with EDs who reported higher levels of childhood teasing and betrayal sensitivity generated more vivid memories and future events in response to disgust-related cue words—a pattern not observed in healthy controls.

**Conclusions:**

Targeted interventions aimed at assessing and mitigating maladaptive disgust responses may be beneficial for individuals with EDs. Findings also inform potential directions for future research, highlighting how refined methodologies across paradigms could further clarify the mechanistic role of disgust in the onset and maintenance of eating disorders.

**Disclosure of Interest:**

None Declared

